# The Mediating Role of Stigma, Internalized Shame, and Autonomous Motivation in the Relationship Between Depression, Anxiety, and Psychological Help-Seeking Attitudes in Multiple Sclerosis

**DOI:** 10.1007/s12529-022-10078-6

**Published:** 2022-03-24

**Authors:** Tamrin Barta, Litza Kiropoulos

**Affiliations:** Melbourne School of Psychological Sciences, Mood and Anxiety Disorders Lab, Faculty of Medicine, Dentistry, and Health Sciences, The University of Melbourne, Melbourne, VIC 3010 Australia

**Keywords:** Multiple sclerosis, Depression, Anxiety, Psychological help-seeking attitudes, Stigma, Shame, Autonomous motivation

## Abstract

**Background:**

Depression and anxiety are commonly experienced in individuals with multiple sclerosis (MS) yet little is known about factors associated with psychological help-seeking attitudes in those with MS.

**Method:**

The current study investigated whether increased stigma related to chronic illness, internalized shame, and autonomous motivation mediated the relationship between depressive and anxiety symptoms and psychological help-seeking attitudes in individuals with MS. Two hundred fifty-four participants with MS completed an online questionnaire assessing depressive and anxiety symptoms, stigma related to chronic illness, internalized shame, autonomous motivation, and psychological help-seeking attitudes.

**Results:**

Stigma related to chronic illness, internalized shame, and autonomous motivation mediated the relationships between increased depressive symptoms and anxiety symptoms and psychological help-seeking attitudes. The study also found that higher levels of chronic illness–related stigma and internalized shame were associated with more negative psychological help-seeking attitudes and higher autonomous motivation was associated with more positive psychological help-seeking attitudes. There were no direct effects of depressive or anxiety symptoms on psychological help-seeking attitudes.

**Conclusion:**

The significant mediating roles of stigma-related chronic illness, internalized shame, and autonomous motivation indicate that these factors may be useful to include in future depression and anxiety intervention studies targeting MS populations.

## Introduction

Multiple sclerosis (MS) is a chronic degenerative central nervous system disease, characterized by demyelinating lesions affecting the brain and spinal cord [1]. Meta-analytic data has shown that the pooled lifetime prevalence of depression in MS populations is high, ranging from 23 to 30%, and is similarly high for anxiety ranging from 21 to 22% [2, 3]. However, research has shown that reported help-seeking for depression and anxiety is low [4]. Psychological help-seeking is an adaptive, intentional process involving a consultation with a healthcare professional to obtain assistance, and understanding psychological help-seeking includes investigating attitudes, behavioural intentions, and observable behaviours [5]. Understanding psychological help-seeking in MS populations would guide psychologists, and other health professionals, in both the assessment process and the development of targeted psychological interventions. To date there is a lack of research exploring mediating factors which impact psychological help-seeking attitudes in MS populations. Research suggests that there may be factors other than depressive and anxiety symptoms which influence psychological help-seeking attitudes and behaviour [6]. Three such mediators include stigma related to chronic illness, internalized shame, and autonomous motivation.

### Psychological Help-Seeking Attitudes

Although there is not a clear, agreed-upon definition of psychological help-seeking, researchers have agreed it is an adaptive process containing attitudes, behavioural intentions, and observable behaviours [5]. Often, these aspects of psychological help-seeking are studied as distinct processes, but evidence has shown that psychological help-seeking attitudes are accurate indications of future observable behaviour. Qualitative research in MS populations has suggested that individuals with MS do not disclose emotional distress to try to cope on their own [7]. Although reasons were varied, generally individuals felt a desire to defy their MS diagnosis and avoid the distress of disclosure often associated with disclosing their MS diagnosis [7]. Longitudinal research has found an association between psychological help-seeking attitudes and future use of mental health services [6]. This association was not significantly affected by previous help-seeking behaviour or the ongoing presence of a mood or anxiety disorder. In other words, the degree of need did not influence the relationship between psychological help-seeking attitudes and actual help-seeking behaviour for psychological distress. In addition, research in non-MS populations has suggested that attitudinal barriers are commonly reported in individuals who recognize a need for treatment [8]. For example, in a survey of 1350 individuals who met criteria for at least one 12-month disorder and who did not use any services, 97.4% of respondents who recognized a need for treatment reported attitudinal barriers which included stigma [8]. It seems plausible that there are other factors which influence and mediate psychological help-seeking attitudes and behaviour in individuals with MS experiencing depression and anxiety. Possible mediators between depression and anxiety include stigma related to chronic illness, internalized shame, and autonomous motivation.

### Chronic Illness–Related Stigma

Chronic illness–related stigma refers to the multi-level process whereby discrimination and stereotypes regarding one’s illness, in this case MS, leads to the personal agreement and self-attribution of these negative stereotypes and discrimination [9, 10]. Chronic illness–related stigma has been found to contribute to clinical levels of depression, increased unemployment and needing a caregiver, and increased feelings of alienation in social and familial relationships in individuals with MS [11–13]. Experiencing at least one psychiatric disorder was significantly associated with increased stigma scores, and strong negative associations have been found between mental health quality of life and stigma [11].

Systematic reviews have shown that mental health–related stigma is associated with reduced help-seeking in the general population [14]. Additionally, research has demonstrated that stigma predicts psychological help-seeking attitudes and willingness to seek therapeutic services in non-MS populations [15]. Considering individuals with MS experience psychological distress at elevated rates, similar findings should exist, or potentially stronger associations, when assessing stigma related to chronic illness as a barrier to psychological help-seeking in individuals with MS.

### Internalized Shame

Internalized shame is also a potential mediator because it has been found to be associated to depression, anxiety, and psychological help-seeking. Shame is a complex emotion, characterized by an individual’s perception that their attributes and behaviours will be negatively evaluated, attacked, and rejected by others [16]. Increased internalized shame, a subcomponent of shame, is related to an increase in the emotional experience of negative self-evaluation and is linked to self-related cognition and affect [16]. The impact of MS on physical capabilities, employment, and social relationships [1, 13, 17] may increase feelings of social unattractiveness, private devaluations, and self-blame [18]. Research has found that interventions for individuals with chronic diseases have positive impacts on both internalized shame and depressive symptoms [19]. However, there is an absence of literature exploring the role of internalized shame in individuals with MS. From the literature available, a significant association has been found between shame, both state and trait anxiety, and depressive symptoms [18, 20]. Moreover, higher levels of shame were found to mediate the relationship between elevated symptoms of depression and anxiety predicting more negative psychological help-seeking attitudes and decreased willingness to seek professional therapeutic help in the general population [21, 22]. Taken together, these findings have demonstrated that shame mediates the relationship between depressive and anxiety symptoms and psychological help-seeking in the general population. It is expected that similar relationships would be found in individuals with MS.

### Autonomous Motivation

According to self-determination theory (SDT), motivation engages a continuum from controlled to autonomous [23–25]. Controlled motivation refers to behaving in response to demands and pressure from external forces, while autonomous motivation relates to self-identified values and the inherent nature to pursue goals and extend individual competencies [23–25]. Research into autonomous motivation in individuals with MS has assessed the impacts of increasing autonomous motivation, through motivational interviewing (MI) techniques, on health-promoting behaviours. Research in this area has shown that MI is an effective tool in increasing health-promoting activities, including mental wellbeing, when compared to controls [26, 27]. These findings provide evidence for a relationship between increasing autonomous motivation and improvements in health-promoting behaviours in individuals with MS.

There is a lack of research investigating the relationship between autonomous motivation and psychological help-seeking in individuals with MS. Recent research has found that increasing autonomous motivation through motivational interviewing in women experiencing depression resulted in the odds for psychological help-seeking being four times higher compared to those women who were depressed in the treatment-as-usual condition [28]. It is plausible that individual differences in autonomous motivation would mediate the relationship between depressive and anxiety symptoms and psychological help-seeking in MS populations.

### The Theoretical Model

Taken together, the available literature has shown stigma, internalized shame, and autonomous motivation are associated with depression and anxiety and act as potential barriers to accessing psychological treatments. Specifically, individuals with MS experiencing mental illness may be exposed to increased experiences of chronic illness–related stigma and shame experiences. Individuals with MS may also differ in their tendency to pursue goals. Moreover, research has shown that addressing self-efficacy and internal goal pursuit have positive outcomes for individuals living with MS [26, 27]. Research suggests that depression and anxiety have a complex origin in MS populations, which include genetic, immunological, and psychosocial factors [29]. However, despite a growing understanding in what may increase depression and anxiety in MS, treatment rates remain low [4]. Qualitative research in an MS population showed that individuals sometimes denied having a mental health problem and acknowledged that shame and stigma experiences may also interfere with their ability and desire to seek help for mental health concerns [30]. It seems plausible that stigma related to chronic illness, internalized shame, and autonomous motivation may influence the likelihood that individuals with MS seek help when experiencing psychological distress.

**Fig. 1:**
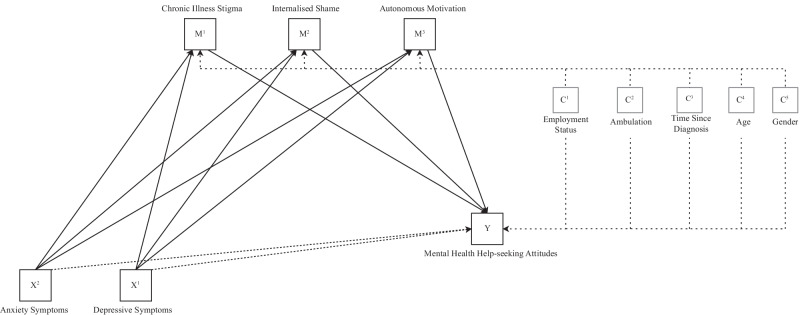
The two proposed parallel mediation models of the present study. Note: The direct relationship of depressive and anxiety symptoms on mental health help-seeking attitudes is shown in black dotted lines. Proposed mediated relationships are shown in solid black lines. Covariates on the relationships between the proposed mediators and mental health help-seeking attitudes are shown in grey. *X* = predictor variable; *M* = mediator; *Y* = outcome variable; *C* = Covariate. Diagram structure per Hayes [41]

We hypothesized that stigma related to chronic illness, internalized shame, and autonomous motivation would mediate the predicted negative relationship between depressive and anxiety symptoms and psychological help-seeking attitudes. Figure 1 displays the proposed mediation models for depression and anxiety and attitudes towards mental health help-seeking in individuals with MS.

## Method

### Participants

We recruited 254 participants with self-reported MS who completed an online questionnaire which was available from June 2017 to June 2020. Participants were recruited through online advertisements published on social media pages of MS-related organizations. Table 1 summarizes the demographic, medical, and clinical characteristics of participants.

**Table 1:** Sample demographic, medical, and clinical characteristics (*N* = 254)

Characteristic	*n*	%	*M* (SD), range
Age			46.04 (12.19), 19–78
Sex			
Male	34	13.39	
Female	220	86.61	
Cultural background/ethnicity			
Australian	115	45.28	
UK	45	17.72	
Asian	3	1.18	
North American	43	16.93	
Eastern European	14	5.51	
Western European	6	2.36	
Southern European/Mediterranean	11	4.33	
African	1	0.39	
Other	16	6.30	
Relationship status			
Single	52	20.47	
Married/living with a partner	155	61.02	
Divorced/separated	30	11.81	
Widowed	2	0.79	
Not living with a partner	15	5.91	
Employment status			
Unemployed	111	43.70	
Part-time or casual	53	20.87	
Full-time	90	35.43	
Antidepressants			
Yes	112	44.10	
Maybe	6	2.36	
No	136	53.54	
Past depression diagnosis	136	53.54	
Past anxiety diagnosis	97	38.19	
Months since MS diagnosis			107 (100.77), 0–684
MS type			
Relapse-remitting	211	83.07	
Progressive	43	16.93	
Current relapse	18	7.09	
Taking MS medication	207	81.50	
EDSS-S classification			
Minimal/absent	162	63.78	
Moderate/high	92	36.22	
DASS-21			
DASS-D			6.69 (5.50), 0–21
DASS-A			4.50 (4.16), 0–17
DASS-S			7.00 (4.92), 0–21
SSCI			51.95 (17.99), 24–106
IASMHS			70.96 (13.46), 27–96
IAF			53.22 (7.11), 35–72
ISS-S			38.52 (23.05), 0–93

*EDSS-S* self-administered Expanded Disability Status Scale, walking distance items; *DASS-21* Depression, Anxiety, and Stress Scales (*DASS-D*, Depression scale; *DASS-A* Anxiety scale; *DASS-S*, Stress scale); *SSCI* Stigma Scale for Chronic Illness; *IASMHS* Inventory of Attitudes Toward Seeking Mental Health Services; *IAF* Index of Autonomous Functioning Scale; *ISS-S* Internalized Shame Scale, Shame subscale

### Measures

Participants completed an online questionnaire which consisted of a total of 133 items and was estimated to take approximately 60 min to complete.

#### Demographic Information

Participants answered 14 questions related to age, ethnicity, gender, education, relationship and employment status, MS diagnosis type, and level of depression and anxiety.

#### Level of Ambulation

The 5-item self-administered Expanded Disability Status Scale (EDSS-S) quantifies the level of disability in individuals with MS [31]. Only the first two walking distance items of the scale were administered and participants were rated as having either absent/minimal or moderate/high ambulation impairment based on these two items. The first two questions were used to assess level of ambulation with these items measuring whether the individual could walk independently and how far they could walk without rest. This method of assessing ambulation in individuals with MS has been previously used in research [33]. The correlation coefficient between patient- and physician-administered ambulation items is 0.89 [31].

#### Level of Depression and Anxiety

The 21-item Depression, Anxiety, and Stress Scale (DASS-21) was used to measure level of depressive and anxiety symptoms in the past week [32]. Each subscale comprises seven items with a 4-point Likert scale ranging from 0 (never) to 3 (almost always) and scores ranging from 0 to 21. Higher scores reflect higher levels of depressive and anxiety symptoms. Only the depression and anxiety scales were used in the current study. Research has found that the depression and anxiety scales have good internal consistency using MS samples (0.95 and 0.85, respectively) [33]. For the current sample, the depression and anxiety scales showed good internal consistency, *α* = .93 and *α* = .82, respectively.

#### Psychological Help-Seeking Attitudes

The Inventory of Attitudes Toward Seeking Mental Health Services (IASMHS) consists of 24-Likert items and three subscales: psychological openness, help-seeking propensity, and indifference to stigma with responses ranging from 0 (disagree) to 4 (agree) [34]. Higher scores reflect more positive attitudes towards mental health help-seeking. Psychological openness, help-seeking propensity, and indifference to stigma have been found to display satisfactory reliability (0.70, 0.76, 0.77, respectively) [35]. The IASMHS showed good internal consistency with the current sample, *α* = .86.

#### Stigma Related to Chronic Illness

The 24-item Stigma Scale for Chronic Illnesses (SSCI) was used to measure both enacted and internalized stigma [10]. The SSCI has a 5-point Likert scale from 1 (never) to 5 (always) and a possible range of scores being 24 to 120. Higher scores relate to greater chronic illness–related stigma. Rao et al. [10] developed the SSCI to measure stigma across neurological conditions including MS populations and displayed high internal consistency (Cronbach’s *α* = .97). The SSCI showed excellent internal consistency with the current sample, *α* = .95.

#### Internalized Shame

Internalized shame was assessed using the Internalized Shame Scale (ISS) [36]. The ISS consists of 30 items evaluating shame and self-esteem with responses ranging from 0 (never) to 4 (almost always). The current study only used the 24-item Shame subscale (ISS-S) for analyses. The ISS-S consists of 24 items on a 5-point Likert scale from 0 (never) to 4 (almost always) which were reverse scored for analysis. The ISS has been found to have high internal consistency (Cronbach’s *α* = 0.97) [37, 38]. The ISS-S showed good internal consistency with the current sample, *α* = .88.

#### Autonomous Motivation

The Index of Autonomous Functioning (IAF) scale was used to assess trait autonomous motivation [39]. The IAF consists of 15 Likert items with responses ranging from 1 (not at all true) to 5 (completely true). Higher scores indicate higher levels of autonomous functioning. The IAF has been found to have good internal consistency, *α* = 0.86 [39]. The IAF showed acceptable internal consistency with the current sample, *α* = .69.

### Statistical Analyses

The data were analyzed using IBM SPSS Statistics (version 26.0). Multi-categorical variables were recoded as dichotomous variables and then dummy coded for correlation and regression analyses. Correlations amongst demographic, medical, and main study variables were assessed using Pearson’s correlations for relationships between continuous variables, phi coefficients for relationships between binary variables, and point-biserial coefficients for relationships between continuous and binary variables. We detected one multivariate outlier (*p* = .0004) when examining values of Mahalanobis distance for significance at *p* < .001 and removed it from the dataset [40]. Additionally, one participant, aged 42 years old, reported 1199 months (99.92 years) since diagnosis, making them an extreme outlier in the dataset and they were removed from further analysis.

Two parallel multiple-mediator models were run through Hayes’ [41] PROCESS macro for SPSS, version 3.5. The PROCESS macro [41] uses ordinary least squares path analysis, with the addition of 5000 bootstrapped samples (percentile method) to determine the significance of indirect effects. Research has shown that the sampling distribution of the indirect effect is often asymmetric, especially with smaller sample sizes [42, 43]. The implementation of bootstrapping solves this problem, through the generation of an approximate sampling distribution for ab [43]. Percentile bootstrapping has reasonable empirical coverage probabilities, close to 95%, when population standardized indirect effects are larger than zero [44]. There is a debate in the literature as to whether using the PROCESS macro [41] or structural equation modelling (SEM) procedures is more robust for use in mediation analyses [45]. However, SEM and PROCESS often generate similar or identical results [41]. This is particularly true for observed variable models, which is the model used in the current research [45].

Model 1 used the DASS-21 Depression scores as the predictor, and model 2 used the DASS-21 Anxiety scores as the predictor. Both models used SSCI, IAF, and ISS-S scores as mediators, and IASMHS scores as the outcome variable. Both models included employment status, level of ambulation, months since MS diagnosis, age, and gender as covariates. The completely standardized indirect effects were used as the measure of effect size, as they are a scale-invariant measure [44]. In pursuing this aim, five variables were included as covariates. Previous research has shown that employment status, level of ambulation, time since MS diagnosis, and age are associated with increased stigma, affective symptoms, and suicidal ideation in individuals with MS [11, 17, 46–48]. Women are also more likely to report and have increased psychological help-seeking attitudes [49, 50]. Thus, we controlled for level of ambulation, months since MS diagnosis, employment status, age, and gender in the mediation models.

## Results

### Preliminary Analyses

There were significant correlations between demographic, medical, and main study variables. For example, age was negatively associated with internalized shame and anxiety scores. Relationship status was negatively associated with stigma related to chronic illness, internalized shame, and depression and anxiety scores. Employment status, months since diagnosis, level of ambulation, and type of MS diagnosis were all negatively correlated with stigma related to chronic illness scores. Additionally, all main study measures were significantly correlated. The mean score, standard deviation, range, and internal consistency for the main study variables, as well as the full correlations amongst the demographic, medical, and main study measures, are shown in Table 2.

**Table 2:**
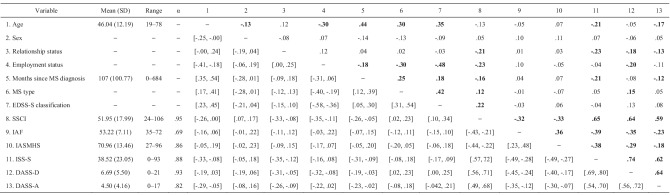
Intercorrelations between the demographic, MS illness, and main study variables, including 95% confidence intervals

Correlations are presented above the diagonal, and associated confidence intervals (CIs) are shown below the diagonal. CIs are based on 5000 bootstrap samples (percentile method). Correlations which have associated CIs that do not cross zero are shown in boldface. Associations between continuous variables were evaluated using Pearson coefficients. Associations between dichotomous variables were assessed with phi coefficients. Associations between continuous and dichotomous variables were assessed with point-biserial coefficients. Dichotomous variables are coded as shown in Table 1

*EDSS-S* self-administered Expanded Disability Status Scale, walking distance items, *SSCI* Stigma Scale for Chronic Illness, *IAF* Index of Autonomous Functioning Scale, *IASMHS* Inventory of Attitudes Toward Seeking Mental Health Services, *ISS-S* Internalized Shame Scale, Shame subscale, *DASS-D* Depression, Anxiety, and Stress Scales (DASS-21), Depression scale, *DASS-A* Depression, Anxiety, and Stress Scales (DASS-21), Anxiety scale

### Parallel Mediation Models

There was no evidence of a direct relationship between state depression scores and attitudes towards psychological help-seeking. The parallel mediation model for depressive symptoms, including regression coefficients and associated 95% confidence intervals (CIs), standard errors, standardized regression coefficients, and model summary information, is detailed in Table 3.

**Table 3:** Model summary information for the parallel mediation model of depressive symptoms on psychological help-seeking attitudes

Model 1 (depressive symptoms)																
	*M*_1_ (SSCI)				*M*_2_ (ISS-IS)				*M*_3_ (IAF)				*Y* (IASMHS)			
	*R*^2^ = 0.47				*R*^2^ = 0.60				*R*^2^ = 0.14				*R*^2^ = 0.24			
	*F*(6, 247) = 36.48, *p* < .001				*F*(6, 247) = 62.23, *p* < .001				*F*(6, 247) = 6.55, *p* < .001				*F*(9, 244) = 8.47, *p* < .001			
	Coef	95% bCI for coef	*SE*	*β*	Coef	95% bCI for coef	*SE*	*β*	Coef	95% bCI for coef	*SE*	*β*	Coef	95% bCI for coef	*SE*	*β*
*X*_1_ (DASS-D)	**1.92**	[1.61, 2.23]	0.16	0.59	**3.15**	[2.81, 3.49]	0.17	0.75	**−0.45**	[−0.60, −0.29]	0.08	**−**0.35	0.13	[−0.31, 0.57]	0.22	0.05
*M*_1_ (SSCI)													**−0.15**	[−0.27, −0.03]	0.06	−0.20
*M*_2_ (ISS-S)													**−0.13**	[−0.24, −0.02]	0.06	−0.22
*M*_3_ (IAF)													**0.44**	[0.21, 0.67]	0.12	0.23
*C*_1_ (employment status)	**−**3.56	[−7.44, 0.32]	1.97	−	1.29	[−3.02, 5.59]	2.19	−	0.46	[−1.49, 2.42]	0.99	−	**−**2.92	[−6.46, 0.61]	1.80	−
*C*_2_ (EDSS-S classification)	**6.27**	[2.27, 10.27]	2.03	−	**−**3.34	[−7.79, 1.10]	**−**2.257	−	0.93	[−1.09, 2.95]	1.02	−	1.83	[−1.91, 5.58]	1.90	−
*C*_3_ (months since MS diagnosis)	**−**0.02	[−0.03, 0.00]	0.01	**−**0.09	**−**0.02	[−0.04, 0.00]	0.02	**−**0.07	0.00	[−0.01, 0.01]	0.00	0.05	0.00	[−0.02, 0.01]	0.01	−0.02
*C*_4_ (age)	**−0.20**	[−0.36, −0.04]	0.08	−0.13	**−0.18**	[−0.36, −0.01]	0.09	**−**0.10	**−**0.05	[−0.13, 0.03]	0.04	**−**0.09	**−**0.02	[−0.17, 0.13]	0.08	−0.02
*C*_5_ (gender)	4.08	[−0.81, 8.97]	2.48	0.08	**5.66**	[0.23, 11.08]	2.75	0.08	1.70	[−0.76, 4.16]	1.25	0.08	**4.64**	[0.15, 9.13]	2.28	0.12
Constant	**46.18**	[36.52, 55.84]	4.90		**23.29**	[12.57, 34.02]	5.45		**56.21**	[51.34, 61.08]	2.47		**57.57**	[40.17, 74.98]	8.84	

Confidence intervals and standard errors were based on 5000 bootstrap samples (percentile method). Coefficients with confidence intervals that do not cross zero are shown in bold. Standardized regression coefficients are unavailable for the dichotomous covariates of employment status

*EDSS-S* classification, and sex, and are denoted with “—.”; *X* predictor variable; *M* mediator variable; *Y* outcome variable; *C* covariate; bCI bootstrap confidence interval; *SE* standard error; *β* standardized regression coefficient. *EDSS-S* self-administered Expanded Disability Status Scale, walking distance items; *DASS-D* Depression, Anxiety, and Stress Scales (DASS-21), Depression scale; *SSCI* Stigma Scale for Chronic Illness; *ISS-S* Internalized Shame Scale, Shame subscale; *IAF* Index of Autonomous Functioning Scale; *IASMHS* Inventory of Attitudes Toward Seeking Mental Health Services. Table structure per Hayes [41]

**Fig. 2:**
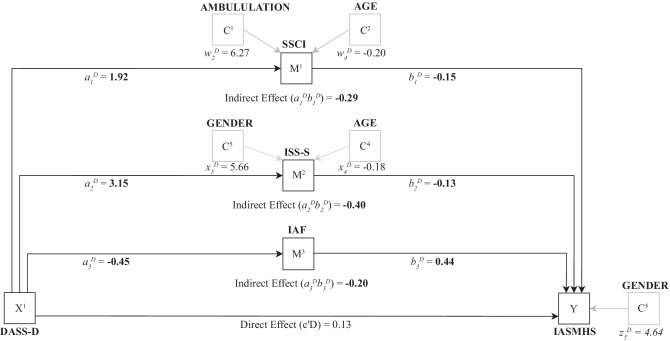
Parallel mediation model of depressive symptoms and attitudes towards psychological help-seeking. Note: Effects with 95% bootstrap confidence intervals which do not cross zero are shown in boldface. Covariates with 95% bootstrap confidence intervals which do not cross zero are shown in grey in the model. Main regression path coefficients are labelled a, b, and c′, where an added D denotes that the coefficient relates to the predictor of depressive symptoms. DASS-D = Depression, Anxiety, and Stress Scales (DASS-21), Depression Scale; SSCI = Stigma Scale for Chronic Illness; ISS-S = Internalized Shame Scale, Shame subscale; IAF = Index of Autonomous Functioning Scale; IASMHS = Inventory of Attitudes Toward Seeking Mental Health Services. *X* = predictor variable; *M* = mediator variable; *Y* = outcome variable; *C* = covariate. Diagram structure per Hayes [41]

The parallel mediation model of depressive symptoms and psychological help-seeking attitudes is shown in Fig. 2.

Of the covariates in the mediation analysis, higher levels of ambulation impairment were associated with higher stigma related to chronic illness scores, as was being a younger age. Additionally, being younger and female were associated with higher Internalized Shame Scores. Finally, being female was associated with more positive attitudes towards psychological help-seeking attitudes. There were three indirect paths from depression to psychological help-seeking attitudes (see Fig. 1): (a) stigma related to chronic illness mediated the relationship between depression and psychological help-seeking attitudes, (b) internalized shame mediated the relationship between depression to psychological help-seeking attitudes, and (c) autonomous motivation mediated the relationship between depression to psychological help-seeking attitudes. For indirect path (a), increased depression predicted higher stigma related to chronic illness which in turn predicted less positive psychological help-seeking attitudes (completely standardized indirect effect coefficient = −0.12, 95% CI [−0.22, −0.01]); for indirect path (b), increased depression predicted higher internalized shame which in turn predicted less positive psychological help-seeking attitudes (completely standardized indirect effect coefficient = −0.16 [−0.31, −0.02]); for indirect path (c), increased depression predicted lower autonomous motivation which in turn predicted less positive psychological help-seeking attitudes (completely standardized indirect effect coefficient = −0.08 [−0.14, −0.03]). The completely standardized total indirect effect from anxiety to psychological help-seeking was −0.36, 95% CI [−0.52, −0.20].

There was no evidence of a direct relationship between anxiety scores and attitudes towards mental health help-seeking. The parallel mediation model for anxiety symptoms, including regression coefficients and associated 95% CIs, standard errors, standardized regression coefficients, and model summary information, is detailed in Table 4.

**Table 4:** Model summary information for the parallel mediation model of anxiety symptoms on psychological help-seeking attitudes

Model 2 (anxiety symptoms)																
	*M*_1_ (SSCI)				*M*_2_ (ISS-IS)				*M*_3_ (IAF)				*Y* (IASMHS)			
	*R*^2^ = 0.41				*R*^2^ = 0.41				*R*^2^ = 0.08				*R*^2^ = 0.25			
	*F*(6, 247) = 29.01, *p* < .001				*F*(6, 247) = 28.80, *p* < .001				*F*(6, 247) = 3.70, *p* = .002				*F*(9, 244) = 8.86, *p* < .001			
	Coef	95% bCI for coef	*SE*	*β*	Coef	95% bCI for coef	*SE*	*β*	Coef	95% bCI for coef	*SE*	*β*	Coef	95% bCI for coef	*SE*	*β*
*X*_1_ (DASS-A)	**2.31**	[1.88, 2.74]	0.22	0.53	**3.33**	[2.78, 3.89]	0.28	0.60	**−0.42**	[**−**0.64, −0.21]	0.11	**−**0.25	0.42	[**−**0.06, 0.90]	0.24	0.13
*M*_1_ (SSCI)													**−0.17**	[**−**0.29, −0.05]	0.06	**−**0.23
*M*_2_ (ISS-S)													**−0.14**	[**−**0.24, −0.04]	0.05	**−**0.24
*M*_3_ (IAF)													**0.43**	[0.20, 0.66]	0.12	0.23
*C*_1_ (employment status)	**−5.43**	[**−**9.47, −1.39]	2.05	−	**−**2.28	[**−**7.46, 2.90]	2.63	−	1.02	[**−**0.97, 3.02]	1.01	−	**−**2.99	[**−**6.49, 0.51]	1.78	−
*C*_2_ (EDSS-S classification)	**5.73**	[1.51, 9.96]	2.14	−	**−**3.80	[**−**9.21, 1.62]	2.75	−	0.95	[**−**1.14, 3.03]	1.06	−	1.71	[**−**2.01, 5.43]	1.89	−
*C*_3_ (months since MS diagnosis)	**−**0.02	[**−**0.04, 0.00]	0.01	**−**0.11	**−**0.02	[**−**0.05, 0.00]	0.01	−0.10	0.00	[0.00, 0.01]	0.00	0.07	0.00	[**−**0.02, 0.01]	0.01	**−**0.03
*C*_4_ (age)	**−**0.12	[**−**0.29, 0.05]	0.09	**−**0.08	**−**0.09	[**−**0.31, 0.13]	0.11	−0.05	**−**0.06	[**−**0.15, 0.02]	0.04	**−**0.11	**−**0.01	[**−**0.15, 0.14]	0.07	0.00
*C*_5_ (gender)	1.28	[**−**3.85, 6.41]	2.61	0.02	1.23	[**−**5.35, 7.82]	3.34	0.02	2.31	[**−**0.23, 4.85]	1.29	0.11	**4.45**	[0.03, 8.87]	2.24	0.11
Constant	**49.07**	[38.97, 59.17]	5.13		**31.74**	[18.78, 44.71]	6.58		**54.60**	[49.60, 59.59]	2.53		**58.27**	[40.94, 75.60]	8.80	

Confidence intervals and standard errors were based on 5000 bootstrap samples (percentile method). Coefficients with confidence intervals that do not cross zero are shown in bold. Standardized regression coefficients are unavailable for the dichotomous covariates of employment status

*EDSS-S* classification, and sex, and are denoted with “−.” *X* predictor variable; *M* mediator variable; *Y* outcome variable; *C* covariate; *bCI* bootstrap confidence interval; *SE* standard error; *β* standardized regression coefficient. *EDSS-S* self-administered Expanded Disability Status Scale, walking distance items; *DASS-A* Depression, Anxiety, and Stress Scales (DASS-21), Anxiety scale; *SSCI* Stigma Scale for Chronic Illness; *ISS-S* Internalized Shame Scale, Shame subscale; *IAF* Index of Autonomous Functioning Scale; *IASMHS* Inventory of Attitudes Toward Seeking Mental Health Services. Table structure per Hayes [41]

**Fig. 3:**
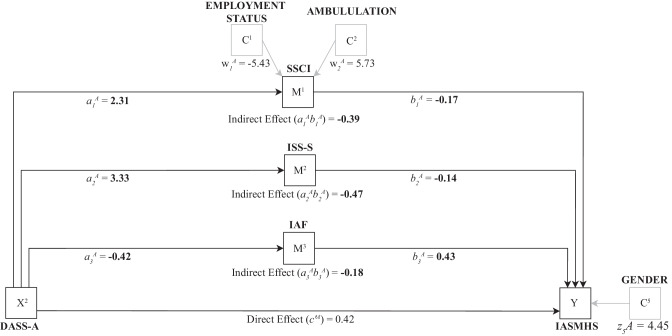
Parallel mediation model of anxiety symptoms and attitudes towards psychological help-seeking. Note: Effects with 95% bootstrap confidence intervals which do not cross zero are shown in boldface. Covariates with 95% bootstrap confidence intervals which do not cross zero are shown in grey in the model. Main regression path coefficients are labelled a, b, and c′, where an A denotes that the coefficient relates to the predictor of anxiety symptoms. DASS-A = Depression, Anxiety, and Stress Scales (DASS-21), Anxiety Scale; SSCI = Stigma Scale for Chronic Illness; ISS-S = Internalized Shame Scale, Shame subscale; IAF = Index of Autonomous Functioning Scale; IASMHS = Inventory of Attitudes Toward Seeking Mental Health Services. *X* = predictor variable; *M* = mediator variable; *Y* = outcome variable; *C* = covariate. Diagram structure per Hayes [41]

The parallel mediation model of anxiety symptoms and psychological help-seeking attitudes is shown in Fig. 3.

Of the covariates in the mediation analysis, being unemployed and higher levels of ambulation impairment were associated with higher stigma related to chronic illness scores. Additionally, being female was associated with more positive attitudes towards mental health help-seeking. There were three indirect paths from anxiety to psychological help-seeking attitudes (see Fig. 2): (a) stigma related to chronic illness mediated the relationship between anxiety to psychological help-seeking attitudes, (b) internalized shame mediated the relationship between anxiety to psychological help-seeking attitudes, and (c) autonomous motivation mediated the relationship between anxiety to psychological help-seeking attitudes. For indirect path (a), increased anxiety predicted higher stigma related to chronic illness which in turn predicted less positive psychological help-seeking attitudes (completely standardized indirect effect coefficient = −0.12, 95% CI [−0.22, −0.02); for indirect path (b), increased anxiety predicted higher internalized shame which in turn predicted less positive psychological help-seeking attitudes (completely standardized indirect effect coefficient = −0.15, 95% CI [−0.25, −0.04]); for indirect path (c), increased anxiety predicted lower autonomous motivation which in turn predicted less positive psychological help-seeking attitudes (completely standardized indirect effect coefficient = −0.06, 95% CI [−0.11, −0.02]). The completely standardized total indirect effect from anxiety to psychological help-seeking attitudes was −0.32, 95% CI [−0.44, −0.20].

## Discussion

Consistent with the hypothesized model, the present study demonstrated that stigma related to chronic illness, internalized shame, and autonomous motivation mediates the relationship between depressive and anxiety symptoms, and psychological help-seeking attitudes in individuals with MS. The current results show an association between higher reported levels of chronic illness–related stigma and internalized shame and more negative psychological help-seeking attitudes. These findings are in line with previous literature examining these relationships in clinical populations and extend them to an MS population [14, 15, 21, 22]. Higher autonomous motivation was also found to be associated with more positive psychological help-seeking attitudes. Additionally, our results did not show a significant direct effect of depressive or anxiety symptoms on psychological help-seeking attitudes. This finding is in line with previous longitudinal research which has shown that the presence of a mood or anxiety disorder does not significantly impact the relationship between psychological help-seeking attitudes and future use of mental health services in non-MS populations [6].

The results of the current study showed that stigma related to chronic illness adversely impacts psychological help-seeking attitudes in MS populations with elevated depressive and anxiety symptoms. These results are consistent with research that has shown a negative association between stigma and psychological help-seeking attitudes and behaviours in non-MS populations [14, 15].

The present study contributes the novel finding that elevated depression and anxiety are associated with increased reporting of internalized shame in an MS population. Research has shown consistent associations between shame and both anxiety and depression [18, 20]. Relatedly, research has also shown that psychological interventions for individuals with chronic diseases have positive impacts on both internalized shame and depressive symptoms [19]. Our results extend these findings to individuals with MS. Moreover, our research shows a relationship between internalized shame and psychological help-seeking attitudes in individuals with MS. Previous research has found an association between the experience of shame and decreased willingness to seek professional therapeutic help [21, 22]. The current research extends these findings by showing that a similar relationship exists with internalized shame in an MS population.

Our findings also show a novel association between higher levels of autonomous motivation and more positive attitudes towards psychological help-seeking in an MS population. This finding is consistent with Holt et al. [28] who found that increasing autonomous motivation resulted in improved odds for seeking help for psychological distress in individuals experiencing depression.

### Limitations

The present study has limitations that should be considered. We used a cross-sectional study design which does not allow us to extrapolate our findings to make causal or temporal claims about the effects of stigma related to chronic illness, shame, and autonomous motivation on the relationship between elevated depressive and anxiety symptoms and psychological help-seeking attitudes [41, 51]. Longitudinal research is required to examine the stability of these relationships in individuals with MS. The present research also utilized self-report for the MS diagnosis with no validation from a neurologist.

### Clinical Implications

Despite these limitations, the present study’s findings have important implications. First, these findings provide support for the assessment of chronic illness–related stigma, internalized shame, and autonomous motivation in individuals with MS who may be experiencing depression and anxiety. These findings show that the experience of chronic illness–related stigma in individuals with MS is associated with depression and anxiety and psychological help-seeking attitudes. Additionally, our results suggest that future research should examine internalized shame longitudinally as a factor associated with depression and anxiety in individuals with MS. Future research and interventions in individuals with MS who are depressed or anxious should focus on targeting feelings of social unattractiveness and self-blame, which are also associated with internalized shame [18]. Third, understanding individual differences in personality which impact individuals with MS would allow for personalized therapeutic interventions. Future research would benefit from investigating individuals’ baseline level of autonomous motivation to improve psychological interventions for depression and anxiety for individuals with MS.

## Conclusion

The unpredictability of the clinical course, the worsening of physical symptoms, and the impacts on valued activities and relationships have substantial psychological effects for individuals with MS [1, 13, 52]. In addition, depression and anxiety are common in MS populations [2, 3, 53], and reported psychological treatment for comorbid depression and anxiety has been found to be low [4, 54]. The present study provides support for the associations between stigma related to chronic illness, internalized shame, and autonomous motivation and depressive and anxiety symptoms and psychological help-seeking attitudes in individuals with MS. Future research should examine these relationships longitudinally and consider these modifiable factors in promoting psychological help-seeking in individuals with MS experiencing depression and anxiety.
